# Supplementary material for: Comparative multiomics analysis of cell physiological state after culture in a basket bioreactor
Source: Sci Rep. 2022 Nov 23;12:20161. doi: 10.1038/s41598-022-24687-4 (PMC9686226; doi:10.1038/s41598-022-24687-4)
Supplement: Supplementary file 1 — Supplementary Information 1. [file 41598_2022_24687_MOESM1_ESM.zip › raw data/Proteomic raw data/1.data_control/dmass_distri.pdf]

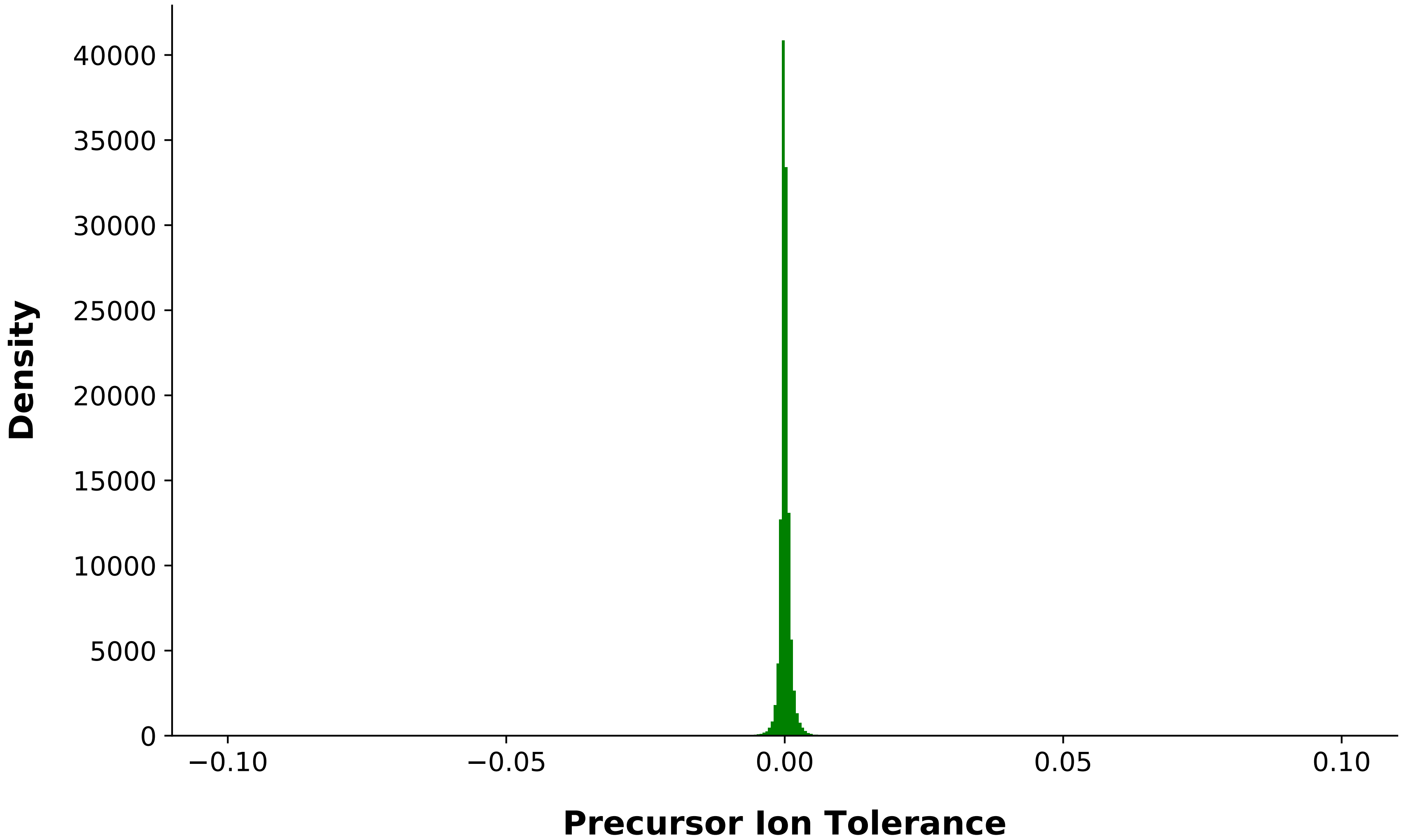

Density
40000
35000
30000
25000
20000
15000
10000
5000
0
−0.10
−0.05
0.00
0.05
0.10
Precursor Ion Tolerance
